# Review of methods for alumina recovery from mudstone and coal fly ash

**DOI:** 10.1016/j.heliyon.2024.e34812

**Published:** 2024-07-19

**Authors:** Amani Shilla, Gershom Mwandila

**Affiliations:** a Chemical Engineering Department, School of Mines and Mineral Sciences, Copperbelt University, Zambia; b Copperbelt University Africa Centre of Excellence (CBU-ACESM), Zambia

**Keywords:** Alumina recovery, Mudstone and coal fly ash, Leaching techniques, Industrialisation, And sustainability

## Abstract

Developing recovery methods from coal mine waste like mudstone and coal fly ash (CFA) is crucial to expanding the alumina supply beyond bauxite. This review explores various approaches for alumina recovery from mudstone and CFA. Six main leaching techniques are discussed—caustic soda, nitric acid, Sulphuric acid, hydrochloric acid, and leaching roasted coal mine wastes. Due to high silica content, these techniques differ from those for bauxite minerals. Alkaline solutions, like sodium and calcium hydroxide, show promise but are cost-intensive. Sulphuric acid, combined with calcium hydroxide or sodium carbonate before roasting, yields efficient results, surpassing 90 % recovery. Microbial extraction also shows promise, but commercialisation faces equipment accessibility challenges. Heat treatment and optimal calcination temperatures are crucial, especially with acid reagents like Sulphuric and hydrochloric acids, preferred for insolubility in silica and better recovery. Sustainable alumina recovery requires further research into economically viable and ecologically friendly technology. This review underscores the need for feasible, high-purity alumina recovery techniques from mudstone and CFA for industrialisation.

## 1 Introduction

The need for mineral and metal commodities has significantly increased due to the constant changes in the world. Consequently, mining, chemical, and other related fields are tasked with finding new sources of minerals and metals, developing efficient methods for extracting them through hydrometallurgical extraction, and creating low-cost chemical refining processes [1]. For example, research into metal recovery from secondary resources such as mudstone and CFA has been motivated by their utilisation [2].Compounds, including alumina, are widely used in many different industries and are well-known for their various properties [3]. It improves oral hygiene by acting as an effective abrasive in toothpaste [4]. Additionally, it can be used as a liner for kilns and furnaces due to its remarkable heat resistance [5]. Alumina improves the mechanical and thermal properties of ceramics, which increases their stability and strength [6]. In addition to being used in pyrotechnics like thermite and rocket fuel [7], alumina is also essential for water treatment techniques. It is used in the production of aluminium sulphate, a compound that is utilised to purify water [8]. In the filtering of drinking water and the treatment of sewage, aluminium sulphate serves as a coagulant. By adding aluminium sulphate, pollutants, organic compounds, and suspended particles are successfully eliminated, enhancing the overall quality and safety of the water [9,10].

As our society becomes more modernised, the demand for aluminium has also increased [11]. Since bauxite ore serves as the primary source of aluminium, there is currently a shortage of high-grade bauxite reserves, leading to significant demand for aluminium [12]. Aluminium is extensively utilised, ranking as the second most used material in the world after steel due to its properties such as strength and lightweight [13]. Its numerous applications across industries such as electronics, automotive, infrastructure, aerospace, and defence have contributed to its steadily rising demand, playing a pivotal role in the global socio-economic advancement [14].

The opportunity is ideal to develop cutting-edge technologies for extracting alumina from both medium and low-grade bauxite ores. China accounted for 53.3 % of the 126.7 million tons of alumina produced globally in 2020. The demand for primary aluminium is projected to increase by 7 % annually worldwide [15]. Therefore, it is crucial to explore alternative sources of alumina, such as mudstone and CFA. Mudstone primarily consists of iron, silicon, aluminium, and other elements, which can be extracted using specific techniques to create innovative products. Gangue typically comprises 40–60 % silica, over 15–35 % alumina, and 1–15 % iron oxide, with the primary goal being alumina extraction [16]. Additionally, the elemental composition of CFA commonly includes trace elements, silicon, aluminium, calcium, iron, magnesium, potassium, and other components. CFA also contains primary oxides such as CaO, SiO_2_, Al_2_O_3_, and Fe_2_O_3_, the quantities of which vary, affecting the characteristics of the fly ash [17]. The composition of CFA can vary based on numerous factors, including the type of coal burned, combustion conditions, and cooling rate [18]. Significant amounts of waste are generated from coal consumption, particularly in countries that heavily rely on coal as a fuel source. China alone has deposited over 4.5 billion tons of coal gangue, while across Europe, 175 million tons of coal waste have been stored in landfills for decades [19]. Globally, billions of tons of CFA are estimated to be produced annually [20]. Over 50 million tons of coal combustion waste are produced annually in South Africa [21]. The exact amount may vary by nation and is influenced by the energy production methods utilised in each region.

Significant amounts of waste are generated throughout the mineral mining process, and these waste materials harm the environment. While some waste generated during mine operations has economic value, there is a significant likelihood that it will persist as a long-term threat and cause recurring environmental harm if not well-utilised [22].As a result, technological advancements in many industries have increased the significance of waste produced by coal mine-related operations [23]. Several techniques for extracting aluminium from mudstone and CFA have been proposed, but no viable technique for commercialisation has been developed yet. The apparent explanation is that the exact content and physical characteristics of such wastes vary depending on the coal source [24].

Coal mine by-products and CFA are potential mineral resources because it is rich in Aluminium and silicon oxide [25]. Coal by-products, such as Mudstone, are produced in the mining sector during the coal cleaning process [26]. Similarly, CFA is just the lighter proportion of non-combustible waste produced from coal combustion in coal-burning electricity generation [27]. Because of its low density, this material is carried away from boilers by flue gases, where it is trapped and collected by gas cleaning equipment such as electrostatic precipitators before the gases are released into the air [28]. CFA and mudstone have traditionally been recognised as contaminants with limited economic potential [29]. Nevertheless, considering the predominance of alumina in these materials, it is interesting to explore their economic application [30]. The accomplishment of such an undertaking would benefit a wide range of sectors. Creating an alternate alumina source from CFA and mudstone would supplement bauxite resources and represent a viable opportunity [31]. However, the separation performance of aluminium oxide and silicon dioxide is insufficient. This inadequacy contributes to the lower utilisation rate of coal waste materials [32]. Alumina-rich coal mine materials have the potential to be a viable source of Alumina [33]. Nevertheless, traditional alumina recovery techniques such as the Bayer process are ineffective in processing low-alumina silica ores because they lead to the simultaneous dissolution of silica and alumina and the precipitation of sodium aluminosilicate hydrate [34]. Numerous studies have been reported on the recovery of alumina from low-grade aluminium sources to address this issue. This technique is focused on mineral processing methods in which acid or alkali is used as the reacting media [35].

Alumina recovery from raw materials in metallurgical processes is adaptable, with methods classified into alkaline and acidic categories that represent various chemical conditions and approaches to optimise extraction from both types of raw materials [36]. With industrialisation, the alkaline method is currently used to create alumina from higher-grade bauxite ore [37]. The caustic dissolves the alumina throughout the ore into the sodium aluminate solutions, but plenty of the iron, titanium, and silicon remain irresolvable [38]. Silica in relatively low bauxite and clay minerals, on the other hand, reacts with alkalis and is transformed into solid waste. As a result, the alkaline method is ineffective for extracting and separating aluminium and silicon at the same time [39]. The acid technique of recovering alumina involves processing an aluminium-containing material using an inorganic acid such as sulphuric acid, hydrochloric acid, or nitric acid to obtain the equivalent aluminium salt solution. The aluminium salt solution is then evaporated or crystallised to produce aluminium salts [40].

The acid method, which involves direct acid leaching [41], high-pressure acid leaching [42], and roasting activation [43], offers a quicker process than the alkali method [44]. This benefit makes it a potential method for separating aluminium and silicon since it helps prevent problems like excessive energy usage and waste residue [45]. Direct acid leaching offers benefits such as lower operational costs, less intensive processing requirements, and reduced energy use, but with lower recovery efficiency. Indirect acid leaching, involving the roasting of mudstone and CFA with or without an alkaline compound at high temperatures before acid leaching, has the disadvantage of increased energy consumption but offers the benefit of achieving higher aluminium recovery efficiency [46]. When acids extract alumina from mudstone and CFA, they dissolve the alumina-containing minerals into soluble forms. The main alumina-containing minerals in mudstone and CFA are aluminosilicates, which react with acids to liberate aluminium ions [47]. During treatment with acids such as sulphuric acid (H₂SO₄), hydrochloric acid (HCl), or nitric acid (HNO₃), the following reactions usually occur: Aluminium oxide reacts with H₂SO₄ to form aluminium sulphate and water [48], with HCl to form aluminium chloride and water [49], and with HNO₃ to form aluminium nitrate and water [45] Since silica (SiO₂) in ores is less reactive to acids than alumina, acid leaching is the preferred method for recovering aluminium. It effectively addresses the issue of high silica content in mudstone and CFA by taking advantage of silica's relative insolubility in acidic media. In contrast, alkaline media can dissolve both silica and aluminium compounds [50].

However, employing the acid technique to remove iron from the leachate is often challenging [51]. Impurity iron can degrade the grade of electrolytic aluminium produced during subsequent electrolysis. Controlling the iron content of the leachate so becomes essential [52]. Iron removal from acid leachate has been accomplished using several techniques, such as chemical precipitation [53], solvent extraction [54], ion exchange [55], and crystallisation [56]. Thorough iron removal is difficult because chemical precipitation frequently necessitates a significant consumption of precipitants and may trap important metals. The tight connection between the chelating group and the ferric ion can make iron stripping challenging and can result in extractant loss during solvent extraction [54]. The ion exchange method offers a straightforward procedure, but because of the resin's relatively low exchange capacity, specific conditions may need to be met to achieve solution stability [57]. Recrystallisation is a difficult process that uses a lot of energy, even though it doesn't add any additives or harm the environment [[58], [59], [60]]. As a consequence, it is essential to develop a method that improves iron removal while extracting aluminium to solve the challenges with impurity control [61].

Bioleaching is a really promising technique that utilises little capital, labor, and energy [62]. It provides an alternate method for boosting the alumina content of CFA and mudstone. This biological process not only has the potential to be ecologically sound, but it also has economic benefits [31]. Substantial tests have demonstrated its efficacy in eliminating various metal contaminants at the same time [63]. Bioleaching has recently developed as a creative approach for extracting metals from a variety of solid industrial wastes [64]. Bioleaching involves the ionisation of cations, which is aided by microorganisms that undertake oxidation/reduction processes. It is critical to identify certain microorganisms capable of ionizing metallic oxides [65]. Aspergillus niger, Penicillium simplicissimum, Penicillium purpurogenum, Rhodococcus rubra, Acidothiobacillus thiooxidans, and Acidothiobacillus ferrooxidans are among the microorganisms typically used in bioleaching [66], and they contribute to both “contact” and “non-contact” Bioleaching technologies. In the contact mechanism, bacteria attach to and absorb bioleaching substrates, whereas the non-contact mechanism uses oxidation reactions including iron (II) ion reduction and sulphur oxidation [67]. Notably, fungi such as Penicillium simplicissimum and Aspergillus niger have played critical roles in the bioleaching of aluminium from bauxite [68].

No process that has been invented has been able to compete economically with the Bayer process. Initially, several of these alternatives were developed as laboratory-scale facilities, progressed to semi-industrial testing, and ultimately were limited to constructing pilot plants [39]. This research aims to enable the industrial-scale recovery of alumina from CFA and mudstone, transforming what was long considered waste into a valuable resource. The paper reviews the extraction techniques currently used for recovering alumina from non-bauxite materials. By applying the knowledge and insights gained from these assessed procedures, it is possible to create alumina recovery systems that are both environmentally friendly and economically viable. As a result, there is less waste and reduced reliance on primary sources of alumina, furthering the ideas of the circular economy. Various research works have been carried out for alumina recovery which is available in the open literature. Table 1 briefly summarises important minerals found in mudstone and CFA, as well as suitable routes for alumina production.

**Table 1:** Alternative sources with potential for alumina extraction.

Non-bauxite sources	Mineral's content	Suitable routes for Al's recovery
CFA	The mineral compositions of CFA can vary depending on the type of coal and combustion conditions. Common minerals found in CFA include quartz, mullite, hematite, magnetite, anhydrite, carbonates, pyrite, chalcopyrite, and ferrospinel. Additionally, ferrospheres may also contain anhydrite, carbonates, sulfides, and copper-iron sulfides in additiontoferrospinel [[69], [70], [71]]	Heat treatment is followed by leaching with water and acid, Direct acid leaching, alkali leaching, and bioleaching.
Mudstone	Typical minerals present in coal gangue or mudstone comprise quartz, kaolinite, montmorillonite, calcite, pyrite, and assorted clay minerals [[72], [73], [74]].	Heat treatment is followed by leaching with water and acid, Direct acid leaching, and alkali leaching.

## 2 Different methods for alumina leaching and recovery from coal mine waste review

**Fig. 1:**
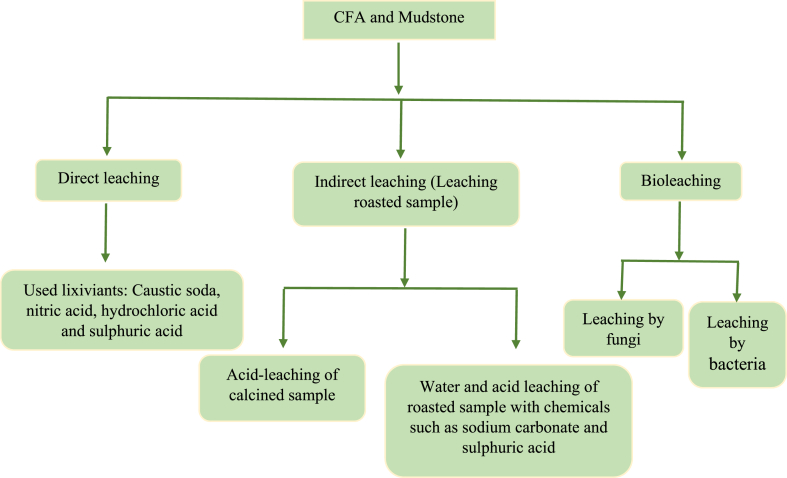
Approaches of alumina recovery from CFA and mudstone.

Numerous methods for recovering aluminium in the literature have been studied, and these methods can be divided into three major categories. Caustic soda, nitric acid, and sulphuric acid are used as leaching agents in the first group where these chemicals work to extract aluminium from multiple sources [75]. Scholars have explored the leaching process in this category utilising caustic soda (sodium hydroxide), nitric acid, and sulphuric acid either separately or in combination [76]. These reviews aim to highlight the approach of optimum leaching conditions and the efficacy of aluminium recovery using these chemical agents. The second category deals with the coal mine waste pre-treatment before the leaching method. Numerous pre-treatment strategies were investigated in these studies to optimise aluminium extraction recovery. The mudstone and CFA are subjected to high temperatures to induce chemical and structural changes. After calcination, the samples are leached using lixiviant agents, exploiting the sample's altered characteristics to maximise aluminium's recovery. The third category is focused on using microorganisms to improve aluminium recovery. The third approach involved forming a microbial community of acidophilic bacteria, fungi, and archaea that survive in highly acidic surroundings. These microbes produce acid by metabolising energy sources such as ferrous iron, sulphur, or hydrogen sulphide, which causes alumina to dissolve under acidic circumstances [77]. The acid reacts with minerals containing alumina, causing it to be released into leachate. The aluminium that has been solubilised is subsequently recovered and purified using different techniques such as precipitation, ion exchange, or solvent extraction [78]. These research areas offer insightful information on various leaching strategies for recovering aluminium, emphasising the significance of choosing suitable leaching agents and considering pre-treatment techniques to increase the process's overall effectiveness. By reviewing and examining these various approaches, as shown in Fig. 1, researchers want to maximise aluminium recovery from coal combustion by-products and mining wastes, aiding in sustainable resource usage and waste management techniques.

### 2.1 Leaching with caustic soda or base solutions

Much literature documented successful results where coal mine wastes such as mudstone, coal gangue, and CFA have been leached with caustic soda for various objectives. H. Li et al. [79] conducted a comprehensive study on recovering alumina from CFA using state-of-the-art autoclaves operating at high temperatures. An advanced control system expertly regulated the autoclave's temperature, agitation, and heating rate. Leaching reagents, namely sodium hydroxide (NaOH) and calcium hydroxide (Ca(OH)_2_), were employed in the experimental setup. During the experiment, the autoclave was filled with CFA, Ca(OH)_2_, and a 40 % NaOH solution. The resulting mixture underwent digestion in the autoclave at varying residence times and temperatures ranging from 230 to 280 °C. The combination of mullite and NaOH during this process produced hydrosodalite, which played a crucial role in facilitating the extraction of alumina from the solution. At higher reaction temperatures, zeolite and NaCaHSiO_4_ formed when hydrosodalite and Ca(OH)_2_ were combined. The zeolite content in the residual materials significantly influenced the alumina extraction ratio. Importantly, the addition of Ca(OH)_2_ to the process was critical in increasing efficiency by preventing silicon from leaching into the solution. According to the study's author, the optimal conditions for alumina extraction were identified at 260 °C, utilising a 40 % weight NaOH solution, maintaining a mass ratio of 1.0 for CaO to SiO_2_, sustaining a liquid-to-solid ratio of 12, and employing a residence duration of 45 min. Under these ideal circumstances, the alumina extraction efficiency reached approximately 91.3 %, with a caustic ratio of 11.5, demonstrating the effectiveness of the proposed extraction method. Similarly, Q. Yang et al. [35] conducted autoclave experiments to extract aluminium from coal by-products rich in alumina, specifically coal gangue. The researchers followed a procedure that involved mixing a sodium aluminate solution with a specific quantity of coal mine wastes, calcium hydroxide (Ca(OH)_2_), while maintaining a molar ratio of sodium oxide to aluminium oxide. Reagents such as sodium hydroxide (NaOH), calcium hydroxide (Ca(OH)_2_), and aluminium hydroxide (Al(OH)_3_) were employed, and specific pressures and temperatures were applied to initiate the extraction reaction. Upon completion of the reaction, the mixture underwent cooling, and the solid and liquid components were separated using filtration. Before undergoing further analysis, the filter residue was thoroughly rinsed with distilled water and dehydrated in hot air ovens. The authors verified that kaolinite could be broken down and converted into sodium aluminium silicate (Na_8_Al_6_Si_6_O_24_(OH)_2_(H_2_O)_2_) and calcium aluminium silicate (Ca_2_Al_2_SiO_6_(OH)_2_) by using NaOH at specific concentrations, in combination with other process parameters such as reaction temperature and time. The investigation's results revealed that the optimal extraction conditions were achieved with a 47.5 % NaOH concentration, an alkali-to-gangue ratio of 6, a reaction temperature of 260 °C, and a reaction duration of 120 min. Under these conditions, the alumina-to-silica ratio of the residue and the aluminium extraction rate reached 0.04 and 94.68 %, respectively. Remarkably, NaCaHSiO_4_ was identified as the predominant phase in the leached residue, making a significant contribution to the successful extraction of alumina from coal gangue.

Furthermore, Cao et al. [80] employed a combination of lime and NaOH to augment the solubility and separation of Al/Si components in a “one-step” hydrothermal process aimed at recovering Al_2_O_3_ from CFA. The study systematically investigated the effects of various variables on the leaching efficiency of alumina, encompassing temperature, reaction duration, NaOH concentration, and the molar ratio of CaO to SiO_2_. Researchers found that when CFA was treated with a mixture of lime and NaOH, the elements of CFA that contained silicon and aluminium underwent enough breakdown. The dissolved silicate ions then combined with the calcium ions to form tobermorite, while the concentrated aluminate ions in the solution led to the specific extraction of alumina from CFA. The results showed that the optimal leaching conditions for Al_2_O_3_ were achieved with an extraction rate of 60.14 %. Specifically, the most effective combination involved the presence of 30 g/L NaOH, a 1.0 M ratio of CaO/SiO_2_, and a 3-h treatment at 210 °C. This underscores the critical role of precisely calibrated parameters in maximising the hydrothermal approach's efficacy in extracting alumina from CFA. The findings contribute valuable insights into the intricate dynamics governing the extraction process and further underscore the potential for refining and optimising the recovery of alumina from coal mine by-products.

### 2.2 Leaching and recovery of alumina by the acidic treatment

#### 2.2.1 Use of nitric acid

Alumina (Al_2_O_3_) present in coal mine waste and CFA can be effectively dissolved using nitric acid (HNO_3_) as a leaching agent. In this process, nitric acid is applied to treat coal mine waste or CFA, prompting alumina to react and form aluminium nitrate, while leaving other undesirable substances unreacted. Depending on the specific procedure employed, the aluminium nitrate can be subsequently separated from the residue using various techniques, such as filtration or precipitation. One notable application of further processing involves converting the aluminium nitrate obtained from the leaching procedure into valuable products, with the conversion to aluminium sulphate (Al_2_(SO_4_)_3_) being a prominent example. In this conversion, aluminium sulphate and nitric acid are commonly produced as by-products through a reaction with sulphuric acid [81]):

2Al(NO_3_)_3_ + 3H_2_SO_4_ → Al_2_(SO4)_3_ + 6HNO_3_

Nitric acid is employed in the leaching process, offering benefits such as selective extraction of alumina by concentrating on its presence while largely disregarding other components. However, it is imperative to note that the use of nitric acid and the by-products generated during the process may necessitate appropriate precautionary measures and considerations for environmental impact. Therefore, it is crucial to take these factors into account when handling and disposing of them [82].

Researchers have employed nitric acid as a leaching reagent in a limited number of studies. X. Li et al. [83] attempted to address this gap by specifically focusing on the process of extracting aluminium from CFA through nitric acid pressure leaching. Upon reaching optimal conditions—220 °C, 300 g/L of nitric acid, a liquid-to-solid ratio of 4 mL/g, and a 2-h duration—they achieved an extraction rate of 84.24 % for aluminium with minimal iron leaching (6.67 %). Analysis of the leachate revealed concentrations of 0.52 g/L for iron and 46.34 g/L for aluminium, signifying successful separation of these elements during the leaching process. The aluminium-rich leachate underwent crystallisation and pyrolysis, with the leach liquor subsequently purified to eliminate remaining contaminants, ultimately yielding Aluminium Oxide (Al_2_O_3_). The primary components of the leach residue were Ferric oxide (Fe_2_O_3_) and silica. In a similar vein, Hou et al. [45] also utilised the nitric acid pressure leaching method in their concerted effort to recover aluminium from CFA. Researchers found that the leachate, with a pH ranging from −0.5 to 1, hindered the formation of Al(OH)_3_ colloids, as per the Al–Fe–H_2_O system's *E*-pH diagram. They identified aluminium mainly as Al^3+^ ions and observed that bivalent iron transformed into hematite after nitrate ion (NO_3_^−^) oxidation. The equilibrium concentration of Fe^3+^ ions (lg cFe^3+^) during Fe^3+^ hydrolysis correlated with pH. Consequently, higher temperatures and acidity led to increased aluminium dissolution in the solution and greater iron retention in the residue. They determined optimal parameters for the method, including a leaching temperature of 220 °C, a liquid-to-solid ratio of 4:1 mL/g, a nitric acid concentration of 340 g/L, and a leaching period of 2 h. These conditions yielded an approximate 80 % leaching rate for aluminium. To facilitate impurity removal, the leachate was concentrated and crystallised in subsequent phases of their research. Notably, aluminium nitrate precipitated at room temperature when the density of the leachate exceeded 1.38 g/cm³. The crystallisation process was aided by further concentrating the leachate to a higher density, resulting in aluminium nitrate crystals with a reduced crystal water content.

#### 2.2.2 Use of sulphuric acid

Many studies have focused on using Sulphuric acid to dissolve aluminium-containing materials. While the use of Sulphuric acid is a common factor across these investigations, the precise procedures vary. For example, in a study conducted by Thamilselvi and Balamurugan [84], CFA underwent leaching with Sulphuric acid at temperatures ranging from 30 °C to 85 °C. The direct leaching process aimed to extract aluminum's amorphous and crystalline phases from the CFA under atmospheric pressure. Maintaining a consistent stirring speed of 200 rpm, the researchers tested various H_2_SO_4_ concentrations (2, 4, 6, 8, and 10 M), leaching durations (2, 3, 4, and 5 h), and solid-to-liquid ratios (1:2, 1:3, 1:4, and 1:6). Subsequently, the mixture underwent extraction using warm purified water and filtration through a vacuum pump via a filter funnel. The residue was rinsed twice with hot distilled water to decrease the acid concentration, resulting in an aluminium recovery efficiency of 94.2 %. Researchers concluded that the efficiency of alumina extraction increases with an increase in the solid-to-liquid ratio. In a parallel study, L-S. Li et al. [85] utilised a mechanical technique to grind CFA samples, which were then combined with concentrated sulphuric acid in a four-mouth flask. An agitator stirred the liquid as it was heated in a heating mantle. The intermediate product, aluminium sulphate, developed on the surface of the leached CFA after excess sulphuric acid was filtered away from the solids. The acid-leaching residue was filtered off, and the aluminium sulphate was dissolved in hot water. They concentrated the aluminium sulphate solution and produced crystals of aluminium sulphate. The researchers identified the ideal conditions for the acid-leaching process as a temperature range of 200–210 °C, a leaching period of 80 min, a volumetric ratio of acid to CFA of 5:1, and an agitation speed of 300 rpm. The extraction efficiency reached 87 %.

These studies illustrate the various strategies employed in the Sulphuric acid leaching of alumina-containing materials. The achievement of effective aluminium recovery depends on specific factors such as temperature, acid concentration, leaching time, solid-to-liquid ratio, and agitation speed. To successfully apply Sulphuric acid leaching in the extraction of aluminium from various waste materials, it is essential to comprehend and optimise these parameters. Moreover, Xu et al. [81] reported an alternative approach involving the use of leaching chemicals (Sulphuric acid and ammonium bisulphate) and autoclave experimentation. Parameters such as mixed compositions of Sulphuric acid (H_2_SO_4_) and ammonium bisulphate (NH_4_HSO_4_), leaching times, temperature, solid-to-liquid ratios, and total hydrogen concentrations were monitored during the experiments, which were conducted on contemporary autoclave. After a thorough investigation of pressure leaching behaviour, the optimal parameters were determined as follows: a liquid-to-solid ratio of 10/4, an AB/SA (ammonium bisulphate/Sulphuric acid) ratio of 1/1, a temperature of 220 °C, and a leaching period of 4 h. The total concentration of hydrogen ([H]), comprising H_3_O^+^, HSO_4_ −, and minor H_2_SO_4_, played a crucial role in alumina extraction within the NH_4_HSO_4_ + H_2_SO_4_ solution. Enhanced alumina extraction efficiency was observed with an increase in the [H] value from 6 mol/L to 15 mol/L. However, further increments beyond this concentration did not significantly enhance extraction efficiency. Thus, it was decided that a [H] value of 15 mol/L was ideal. When utilising various sources of CFA, the alumina extraction efficiency under these defined conditions consistently exceeded 87.5 %.

#### 2.2.3 Use of hydrochloric acid

Hydrochloric acid is an effective agent for leaching coal gangue and CFA, resulting in the formation of aluminium chloride, which may then be converted into aluminium sulphate through a reaction with sulphuric acid. The use of hydrochloric acid extends beyond this specific use since it is frequently used in other mining operations for activities such as ore treatments, metal recovery, separation, purifying, and treatment of water [86]. In a lab-scale experiment conducted by Valeev et al. [87], aluminium leaching from CFA was facilitated using a high-pressure autoclave system. The experiment employed stainless steel containers with Teflon-coated inserts, and the autoclave heat was precisely regulated. Various stages, including humid separation, milling, screens, froth, autoclave dissolution, and filtration, were involved and exposure times ranged from 1 to 4 h at temperatures of 170, 180, 190, and 200 °C. Hydrochloric acid at a concentration of 345 g/L (30 %) was used, and the solid-to-liquid ratio (S:L) varied from 1 to 3–7. The obtained residue underwent cleaning and examination before conducting water treatment studies using river water. The water treatment process involved a short-term sedimentation approach, measuring turbidity, and analysing chromaticity, aluminium, iron, and permanganate indexes. The coagulation investigation was carried out in three stages—mixing, coagulation, and precipitation. The coagulation efficiency was determined by analyzing levels of aluminium, iron, and permanganate in filtered water, ensuring compliance with environmental regulatory limits. The study revealed that high-pressure autoclave leaching with hydrochloric acid achieved an efficiency of 95 % at 210 °C, with an ideal HCl concentration of 30 %, a solid-to-liquid ratio of 1:5, and a duration of 3 h.

Cui et al. [88] utilised hydrochloric acid in leaching tests for aluminium extraction from coal mine waste (CMW) within a thermostatically controlled water bath. Solution samples obtained at different time intervals were analysed to determine the dissolving percentages of aluminium oxide (Al_2_O_3_). The goal of the experiment was to assess how temperature and acid concentration affected aluminium oxide extraction. Two main chemical reactions occurred during the complex process of leaching CMW in HCl solution: aluminium oxide reacted with hydrochloric acid to form aluminium chloride and water, while iron (III) oxide reacted with hydrochloric acid to form iron (III) chloride and water. The findings revealed that the optimal range of temperatures for Al_2_O_3_ extraction was between 90 °C and 106 °C, and the ideal concentration of HCl for recovering Al_2_O_3_ was 6 mol·L-1. Al_2_O_3_ recovery from CMW was 94.5 %, and this process effectively supports the recovery of high-quality aluminium. Additionally, researchers have proposed that enhancing the economic feasibility of various leaching techniques requires the development of efficient fine-grinding technology.

Cheng et al. [89] are another group of researchers who focused on extracting aluminium from coal mine waste (coal gangue) by leaching it with hydrochloric acid. The leaching process was conducted at 105–108 °C, and the solid and liquid phases were separated by vacuum filtering for further analysis. Based on the analysis of the reserved filtrate, the percentage extraction of aluminium was determined to be beyond 70 % under the following optimal conditions: an S:L ratio set at 0.33, a leaching time of 3.5 h, and HCl usage at 112.5 mL for a 50 g sample. To improve the extraction of Al_2_O_3_, researchers also introduced sodium fluoride (NaF) to coal waste before calcination and leaching. NaF facilitated the breakdown of the SiO_2_–Al_2_O_3_ bond in the coal waste, resulting in the extraction of over 90 % of the Al_2_O_3_.

Furthermore, by employing hydrochloric acid and Sulphuric acid as leaching agents, Kong et al. [52] conducted a study on the recovery of alumina from coal gangue. Initially, coal gangue powders underwent a 2-h calcination process at various temperatures. Subsequently, leaching occurred at a solid-to-liquid ratio of 1:3.5, utilising 3.0 mol/L Sulphuric acid and 6.0 mol/L hydrochloric acid at a stirring temperature of 93 °C. Following filtration, washing, and drying of the resultant solid and liquid blends, the residual yields were determined. In the situation where the gangue powder was finer than 160 mesh, the calcination temperature was set at 675 °C with a duration of 60 min. The calcined coal gangue was then subjected to leaching with 6 mol/L hydrochloric acid at 93 °C for 4 h. Under these specified conditions, the leaching ratio of iron ions reached 91 %, while that of aluminium ions achieved 66 %, demonstrating successful aluminium extraction. Additionally, Al-Harahsheh et al. [90] conducted an investigation in which HCl was used as a leaching agent to recover aluminium from kaolin. Their investigation focused on the effects of different leaching factors, such as the molarity of the leaching reagent, leaching temperature, and leaching time. Interestingly, their results showed that aluminium could be completely extracted from kaolinite at temperatures higher than 180 °C and leaching times of 120 min. These findings clearly show a favourable correlation between higher temperatures, and longer leaching times, and the recovery of aluminium and other elements. In terms of alumina recovery, the researchers concluded that hydrochloric acid performed substantially better than sulphuric acid.

### 2.3 Leaching roasted coal mine waste and CFA sample

This technique entails blending coal-related waste with specific chemical compounds, followed by exposure to heat. The mixture is subsequently crushed and leached with water and lixiviant agents like sulphuric acid, nitric acid and hydrochloric acid. This method has led to a notable increase in recovery rates by utilising chemicals to dissolve coal mine waste, facilitating the breakdown and dissolution of the alumina complex. This step is crucial for extracting the required components from the samples. The dissolved solution is subsequently transferred to a high-temperature oven known as a muffle furnace. Roasting occurs at different temperatures, subjecting materials to controlled changes that affect their composition and properties. After calcination, the resulting material is crushed, reducing it to fine particles or powder form. This pulverisation process increases the surface area of the samples, facilitating subsequent leaching treatments [91]. Following the crushing of the mudstone, the desired components are extracted using water and hydrochloric acid. Water is commonly utilised as a neutral solvent, whereas hydrochloric acid is employed due to its ability to dissolve and solubilise specific components [92]. Although this method has shown great success rates, meaning that a high percentage of the target components is recovered, it is not without downsides. The consumption of energy and resources during roasting, potential environmental repercussions from the use of sulphuric acid and hydrochloric acid, the generation of waste by-products, and the overall complexity and cost of the process are among the negatives [93].

Scholars have conducted several investigations to enhance the extraction rate using a two-step acid leaching procedure that includes roasting. Zhu et al. [94] performed the first leaching step by leaching CFA with sulphuric acid at 120 °C for 2 h and a solid-to-liquid ratio of 1:2. The initial leaching residue and leachate were obtained, and the first aluminium leaching rate was calculated. The undissolved aluminium residue was then combined with sodium carbonate and roasted in a muffle furnace at 860 °C for 2 h. The amount of sodium carbonate utilised was determined by the amount of SiO_2_ contained in the fly ash leaching residue, with a 1:1 M ratio of Na_2_CO_3_ to SiO_2_ maintained. The roasted product was then leached with water, which dissolved the majority of the sodium silicate but left aluminium in the water-leaching residue. Finally, at temperatures ranging from 20 to 100 °C, sulphuric acid was used to leach the remaining aluminium in the water-leaching residue. Filtration was used to recover the second leachate and leaching residues, and the second aluminium leaching rate was determined. The overall aluminium extraction efficiency was calculated, and the two-step leaching method resulted in high aluminium dissolutions (97.68%–99.83 %). These findings revealed that aluminium and silicon may be effectively separated from coal ash.

Similarly, Shemi et al. [95] used a thermal reciprocal shaking bath and a filter funnel with filter paper to research a two-step acid-leaching procedure. At an initial temperature of 82 °C, with a leaching period of 10 h, an acid concentration of 6 M, a solid-to-liquid ratio of 1:4, and an agitation rate of 150 rpm, raw CFA was leached without any pyrometallurgical pre-treatment. After leaching, the CFA residue and leach liquid solution were sifted, and the residues were rinsed with distilled water and finally dehydrated before further processing. The residual and filtrate were analysed to determine their composition. The CFA residual from the initial leaching process, powdered coal, and calcium carbonate were combined with water to create spherical pellets. In the second stage, the CFA residual from the initial leaching process, powdered coal, and calcium carbonate were combined with water to create spherical pellets. The granules were then roasted for 2 h in a muffle furnace at 1150 °C to produce a sinter product for the second leaching stage. After cooling and crushing, a portion of the sintered pellets was examined using X-ray Diffraction and X-ray Fluorescence. The pellet powder was subjected to the second leaching technique and conditions described above. After leaching, the slurry mixture was filtered, and the residue was washed and dried. Subsequently, both the filtrate and residue underwent evaluation. The cumulative extraction rate for aluminium in the initial and subsequent leaching processes was 88.2 %. This study demonstrates the usefulness of two-step acid leaching methods in enhancing aluminium recovery rates, with high dissolution percentages.

To enhance the efficiency of aluminium leaching, the indirect acid-leaching process usually involves the roasting of CFA and coal mine waste (Mudstone) before they are dissolved in acid. Previous research has demonstrated that low-temperature acid leaching is ineffective for recovering aluminium from coal mine waste and CFA. As a result, thermal activation is frequently used as a pre-treatment to improve mineral reactivity and boost the efficiency of aluminium extraction. Researchers have concentrated on transforming the mullite phase into a more soluble form. Kelmers et al. [96] developed a method for elevating aluminium recovery from CFA by roasting it with a CaSO_4_–CaCO_3_ flux before leaching. Sintering, which takes 3 h at 1450 °C, converts alumina to an acid-soluble phase. The resultant product is then leached in two stages over 6 h, yielding an overall aluminium extraction efficiency of 98 %. Aluminium, iron, and titanium leach together in the first stage. The pH of the leaching liquid is adjusted to allow solvent extraction of iron and titanium. In the second stage, a residue lacking in other metals is leached. The leaching liquid contains aluminium sulphate (Al_2_(SO4)_3_), which reacts with ammonium sulphate ((NH_4_)_2_SO_4_) to precipitate alum (NH_4_Al(SO_4_)_2_), which is then preheated to achieve the expected alumina product. Sibanda et al. [97] summarised an indirect alkaline leaching procedure used in several investigations. The sintering of a mixture of CFACaCO_3_, sulphur, and carbon at roughly 1200 °C results in the creation of calcium sulfo-aluminate clinker. Following that, the clinker is treated with a 3-wt percent Na_2_CO_3_ solution at 65 °C, resulting in aluminium dissolution and a stated alumina recovery rate of 90 per cent. In addition to this procedure, other researchers have investigated the lime sinter process, an alternate indirect alkaline leaching option. CFA is mixed with lime, often in the form of limestone, and heated to 1100 °C in this procedure. This temperature causes calcium aluminate (12CaO7Al_2_O_3_) and di-calcium silicate to develop (2CaOSiO_2_). Once cooled to temperatures below 500 °C, the sinter spontaneously disintegrates, shattering into fine powder as a result of phase transitions and volume expansion during cooling. This eliminates the requirement for sinter grinding before leaching. In leaching reagents such as Na_2_CO_3_, the produced calcium aluminate (12CaO7Al_2_O_3_) is soluble, whereas di-calcium silicate (2CaOSiO_2_) stays insoluble. Low-concentrated alkaline solutions, such as sodium hydroxide and water, can also be used as leaching agents. After leaching, aluminium is present in the pregnant liquid in the state of sodium aluminate, while the majority of silica stays solid as dicalcium silicate. However, some silica co-dissolution may occur, contaminating the NaAlO_2_ liquid and prompting a desilication process. Desilication was accomplished by mixing the pregnant solution with Calcium hydroxide at 70 °C, resulting in the creation of calcium aluminosilicates with poor alkaline solubility. The purified NaAlO_2_-rich solution carbonizes when it comes into contact with CO_2_ bubbles, resulting in the precipitation of Al(OH)_3_. After that, the precipitate is calcined to obtain the final alumina product.

### 2.4 Leaching of alumina from CFA by using of microorganism

The exploration of bacterial microbes for optimising aluminium extraction from coal-produced waste has captured the attention of numerous scientists. This innovative technology draws inspiration from the successful processing of refractory ores associated with various metals, including silver, uranium, copper, and gold. Over the past few years, several studies have extensively documented the bioleaching of diverse materials, such as kaolin, bauxite, and low-grade bauxite, employing microbial agents like Aspergillus niger, Penicillium simplicissimum, Penicillium purpurogenum, Rhodococcus rubra, Acidothiobacillus thiooxidans, and Acidothiobacillus ferrooxidans [97]. In the context of alumina recovery from CFA, the utilisation of specific fungi and bacteria is discussed below.

#### 2.4.1 Leaching by bacteria

Sen et al. [67] tested four distinct microorganisms for bioleaching CFA: Bacillus barbaricus, Aspergillus niger, Penicillium simplicissimum, and Pseudomonas putida. Bacillus barbaricus had the most efficacy in removing silica from CFA, outperforming the other three typical bacteria by a fold of 60. Bacillus barbaricus was identified as a promising bacterium for leaching silica from fly ash, a process that concentrates alumina inside the CFA. These findings show that bioleaching could be a viable approach to enhance the alumina content of CFA. The study found that nutrient combination (such as galactose, tryptone, sodium dibasicphosphate, and sodium chloride), pH (6), temperature (37 °C), CFA concentration (1 per cent with a particle size of 240 mesh), and inoculum concentration all affected the efficiency of bioleaching CFA using Bacillus barbaricus (40 %). Under the conditions above, Bacillus barbaricus was identified as the microorganism to be used for subsequent CFA leaching procedures.

Fan et al. [98] used Acidithiobacillus ferrooxidans as the microbial agent in another study. CFA was mixed in various ratios with sodium carbonate before bioleaching. The mixture was then roasted for 2 h at a temperature of 850 °C, converting the Al-bearing mullite and sillimanite into recoverable nepheline (NaAlSiO_4_). This change improved the accessibility of alumina and other metals in the CFA for bioleaching. The study discovered that the optimal sodium carbonate to CFA activation ratio was 1:0.22, resulting in approximately 80 % aluminium recovery via Na_2_CO_3_ leaching. Thus, roasting with Na_2_CO_3_ before bioleaching plays a critical role in the recovery of alumina and other metals from CFA by substantially elevating extraction rates. Moreover, the authors studied aluminium extraction from roasted CFA via bioleaching with meso-acidophilic Acidithiobacillus ferrooxidans in the presence of pyrite. The study recovered 91.21 percent aluminium and 63.4 percent cerium under conducive leaching conditions: a pyrite/a-CFA mass ratio of 1.5:1, a pulp density of 20 g/L, an initial pH of 1.75, and an initial inoculum of 3 × 10^8/mL. Process parameters such as nutrient combinations, pH, temperature, and inoculum concentrations have been studied to improve bioleaching efficiency. Furthermore, pre-treatment techniques like roasting with sodium carbonate have been observed to improve aluminium and other metal extraction rates from CFA. These findings lead to the development of more sustainable and effective ways for extracting valuable materials such as alumina from CFA andmudstone.

#### 2.4.2 Leaching by fungi

Fungi, as reported by other experts, excel in recovering metals, including alumina, from CFA. Meer and Nazir [99] provided literature on the utilisation of Aspergillus niger in both one-step and two-step procedures. The two-step method involved pre-culturing the fungus for variable lengths of time before incubation with fly ash, while the one-step procedure involved directly incubating the fungus with ash without any preculturing time The two-step procedure often demonstrated better bioleaching than the one-step method due to increased spore germination and optimized pulp densities. Pre-treatments like water washing (WW) have proven helpful in eliminating water-soluble salts, thereby increasing the efficacy of the bioleaching (BL) process. Furthermore, it was found to be vital to optimise several variables, such as pH, fly ash pulp density (FAD), solid-to-liquid ratio (S/L), sucrose concentration, inoculum spore concentration, shaking speed, and the time of fly ash addition to the fungus. Increased leaching efficacy resulted from performing these optimisations at the proper rotational speed (100–140 rpm) and temperature (30 °C). The authors recorded the following reaction parameters as the best for recovery: 100 mL sucrose, 1 % w/v FAD, 14 days of preculturing, 30 °C, 120 rpm, and S/L (1 g 100 mL-1). The leaching method, with over 80 % efficiency, was found to be most effective under these conditions for efficient metal recovery.

## 3 Purification of pregnant leaching solution

The quality of alumina can be negatively impacted by impurities, particularly iron, titanium and silica, present in the solution obtained from the previously stated procedures, as noted by Sonthalia et al. [100] as such, one critical factor in guaranteeing the quality of aluminium goods is the separation process. Magnetic separation is the most commonly employed method, according to X. Li et al. [83] for removing iron from CFA raw materials. However, because the minerals in the CFA differ in terms of their magnetic susceptibility and strength, the technique's efficacy can vary greatly. For this reason, it's important to choose a method that is compatible with the CFA's particular mineral composition. A range of approaches are employed in the pursuit of eliminating contaminants such as titanium, iron and silicafrom leachate, hence providing multiple pathways for purification [101]. Prominent researchers' studies have bolstered these processes, which include precipitation, solvent extraction, ion exchange, and recrystallisation. In this field, Hu et al. [102], Liang et al. [103], L. Wang et al. [54] have all made significant contributions. When employed as a whole, these studies demonstrate the many approaches taken to enhance leachate, which facilitates the removal of hazardous substances and improves the quality of alumina extraction.

While each of these approaches has specific benefits, it also has drawbacks. Although precipitation is frequently used to remove iron, there are potential side effects, such as co-precipitation and unintentional absorption of important metals [104]. Whereas solvent extraction is known for its effectiveness and selectivity, it frequently requires many extraction steps to maximise iron separation and requires the use of organic reagents [105]. Conversely, re-crystallisation can provide a product that is quite pure, but it can also cause a large quantity of aluminium to be lost throughout the process [106]. Ion exchange works incredibly well for removing low-concentration iron ions, but when dealing with high-concentration iron ions, it presents capacity and cost issues [107]. Given their potential to introduce additional elements into the solution, methods that include the addition of oxidants to oxidise, and remove iron should be used sparingly [108].

Among such techniques, polymerisation with calcium aluminate powder produces polyaluminium chloride, which is a straightforward and economical way to use the solution [109]. This adaptable product serves a variety of industries, including the chemical industry and water treatment facilities. It can be applied immediately in solution form or dried for use as a water purification agent [110]. Additionally, it is an important precursor for the crystalline aluminium chloride that is produced and used in additives, casting, medicines, and other industrial settings [111]. To produce high-purity alumina, several crystallisation techniques can be used, which will ultimately increase the effectiveness of resource utilisation and reduce waste [112].

The production of high-purity ammonium alum crystallised from solutions containing impurities was studied by You et al. [113] they produced aluminium sulphate solutions, which also removed contaminants, by digesting metallurgical aluminium hydroxide with Sulphuric acid. They added ammonium sulphate to the prepared aluminium sulphate solutions by the theoretical molar ratio of ammonium sulphate to aluminium sulphate. Following the complete dissolution of the ammonium sulphate, the solutions were filtered. Even yet, there were still contaminants in the final clean solution. To solve this, a crystallisation procedure was performed without agitation utilising a magnetic stirrer and a jacketed glass crystalliser. Using a vacuum freeze dryer, the resultant ammonium alum crystals were dried. Based on their research, the researchers concluded that, in terms of purity and crystal quality, non-agitation crystallisation is preferable to agitation crystallisation. Slower growth rates were reported in the absence of agitation, which were mainly controlled by diffusion processes as opposed to surface integration. Because it gave contaminants less time to integrate into the crystal lattice, this slower growth rate helped produce crystals with higher purity. Essentially, the writers emphasised how crucial it is to carefully regulate the crystallisation process to produce high-purity crystals. This exacting control includes the strategic use of seed crystals to govern crystal growth as well as the optimisation of several parameters, such as supersaturation, temperature, and agitation.

As well, Sangita et al. [114] concentrated on using precipitation to remove impurities from fly ashes from thermal power plants to recover aluminium as aluminium sulphate following acid leaching. They used aluminium sulphate precipitation to eliminate contaminants. After filtering and washing, the leached liquid was collected and allowed to gradually evaporate, concentrating the liquor until aluminium sulphate started to precipitate. Following an acetone wash, the precipitate was vacuum-desiccated to finish drying it. Due to its high hygroscopicity, aluminium sulphate was first dried at 60 °C and then kept in a desiccator until it was subjected to physico-chemical examination. The purity of aluminium sulphate recovered from CFA was not less than 82.42 %, as the paper's conclusion noted.

Other researchers have successfully employed solvent extraction to purify alumina leach solutions. Notably, Rampou et al. [115] performed a purification of CFA leach liquor using an integrated solvent extraction and precipitation approach to remove titanium (Ti) and iron (Fe). Their solvent extraction experiments included a variety of factors such as contact time, aqueous-to-organic ratio, and extractant concentration. They established effective purification methods, such as reducing Fe³⁺ to Fe^2^⁺ before solvent extraction to avoid co-loading with Ti⁴⁺. Using 10 % Primene JMT in kerosene resulted in high extraction efficiency for Ti⁴⁺ and other metals. Optimal results were found at a 1:1 aqueous-to-organic ratio and a 15-min contact time. Furthermore, the crystallisation of Al³⁺ and the precipitation of Fe^2^⁺ as Fe(OH)₂ were significant in alumina recovery and impurity removal. Sun et al. [116]also had significant achievement in their effort to remove ferric ions from aluminium solutions using solvent extraction. To successfully separate ferric ions from aluminium solutions, they developed a novel technique that uses di-(2-ethylhexyl) phosphoric acid (P204) and a tertiary amine compound in a sulfonated kerosene diluent. Their research identified the optimum factors for extraction and stripping, which included a reaction time of more than 20 min, an initial pH range of 1.5–2, a 1:1 phase ratio, and room temperature. These conditions resulted in the removal of more than 97 % of ferric ions, with a recommended 5-min residence time for complete removal in a counter-current operation. Furthermore, the researchers demonstrated over 99 % stripping efficiency using 1 mol/L sulphuric acid, which proved to be an important step in restoring the extractant's efficacy. In contrast, Wang et al. [117]developed a sustainable method for efficiently extracting Fe(III) from CFA sulphuric acid leach liquid, using 2-ethylhexyl phosphonic acid mono-2-ethylhexyl ester (P507) as the extractant and dichloromethane as the diluent. Their research demonstrated P507's higher extraction selectivity towards Fe when compared to P204, underscoring its potential for widespread use. Their results indicated that over 97.6 % of Fe(III) could be efficiently removed under optimal conditions, confirming P507's promising performance and long-term reusability. After extraction, Fe-loaded P507 underwent a stripping process to regenerate and reuse the solvent, with the stripping efficiency investigated with various solutions. The use of hydrogen peroxide, along with H_2_SO_4_, as a stripping agent, was efficient in removing Fe and Ti from the loaded P507, allowing for regeneration and reuse. This process provides a long-term solution for removing Fe from alumina leach solution, resulting in the recovery of precious metals.

## 4 Summarised literature

**Table:** 

	Key aspects of literature	Similarities	Reference
1	The exceptional reactivity of sodium hydroxide has resulted in good recovery rates when leaching CFA with caustic soda (NaOH). Caustic soda, as a strong alkaline agent, may effectively react with the various constituents of CFA and coal mine waste (Mudstone). Its highly reactive nature aids in dissolving important components such as alumina (Al_2_O_3_) and silica (SiO_2_), which are the main constituents of CFA.	Agreement with H. Li et al. [79], Cao et al. [80], and Shoppert et al. [118] where NaOH and Ca(OH)_2_ were used.	[79,80,118]
2	Recent research suggests nitric acid is effective for selectively recovering aluminium from CFA. Nitric acid pressure leaching achieved remarkable results, with one study achieving 84.24 % aluminium extraction and minimal iron leaching. Aluminium (46.34 g/L) and iron (0.52 g/L) were effectively separated. Purification yielded aluminium oxide (Al_2_O_3_), while silica and Fe_2_O_3_ comprised the main leach waste. Another study achieved an 80 % leaching rate for aluminium using concentration and crystallisation to remove impurities.	Agreement with Hou et al. [45] and X. Li et al. [83]	[45,83]
3	Sulphuric acid leaching of coal mine waste and CFA yields notable recovery rates, especially for alumina. Its strong acidic nature facilitates vigorous reactions with various components present. This acidity aids in dissolving alumina (Al_2_O_3_), enhancing efficiency. Leaching can be conducted at ambient or elevated temperatures to improve reaction kinetics and overall efficacy.	Agreements with Thamilselvi and Balamurugan [84],L.-S. Li et al. [85], Meng et al. [119], and L. Wang et al. [54] where sulphuric acid was utilised as leaching reagents.	[52,54,84,85,119]
4	Hydrochloric acid efficiently leaches coal mine wastes and CFA, yielding aluminium chloride. Upon reaction with sulphuric acid, this chloride transforms into aluminium sulphate. Initially, the leaching process treats coal mine wastes with hydrochloric acid, reacting with aluminium oxide (Al_2_O_3_) to produce soluble aluminium chloride (AlCl_3_). Optimized parameters such as temperature and acid concentration enhance alumina extraction efficiency. Subsequently, aluminium chloride obtained from leaching undergoes treatment with sulphuric acid to yield aluminium sulphate (Al_2_(SO4)_3_), a versatile compound widely used, including in water purification.	Agreement with Cheng et al. [89], B. Peng et al. [86], and Cui et al. [88] where hydrochloric acid was used as a Leaching reagent.	[[86], [87], [88], [89]]
5	Heat treatment and roasting of coal mine waste and CFA, followed by a two-step acid leaching method, substantially improve alumina recovery. This approach removes impurities, induces structural changes, and reduces organic carbon content. Acid leaching, utilising solutions such as sulphuric acid or hydrochloric acid, selectively dissolves alumina. This combined treatment enhances the ash's accessibility and reactivity, facilitating efficient alumina extraction.	Agreement with Xie et al. [120], Zhu et al. [94], Shemi et al. [95], and Kelmers et al. [96] where the experiment involved heat treatment before leaching	[[94], [95], [96],^120^]
6	The utilisation of bacterial microorganisms in bioleaching techniques appears to be promising for enhancing aluminium recovery from CFA. Microbial agents such as various fungi and bacteria have been studied for their efficiency in removing silica and leaching alumina and other metals from coal mine waste and CFA. These bacteria possess enzymatic skills that allow them to break down minerals and release important components, improving access to alumina by specifically targeting silica and resulting in enhanced recovery.	Agreement with Sen et al. [67], Sibanda et al. [97], and Fan et al. [98] on the microorganism utilisation for alumina recovery	[67,98]

## 5 Discussion of the summarised literature

Alumina recovery from coal mine waste and CFA is achievable through diverse leaching methods utilising reagents like caustic soda, hydrochloric acid, Sulphuric acid, nitric acid, and microbial agents in bioleaching processes. Each method comes with its own set of advantages and drawbacks that necessitate careful consideration. Notably, this approach cannot adhere to the Bayer process due to its high silica content. Caustic soda leaching, which efficiently recovers alumina from coal mine waste and CFA, offers a rapid rate of recovery. This technique enables the selective dissolving of aluminium while retaining contaminants. However, its considerable expense presents economic challenges at the industrial level. Despite its potential for effective extraction, alternative leaching reagents are often favoured for economic reasons. Acid leaching, which uses reagents such as hydrochloric acid, Sulphuric acid, and nitric acid, is a cost-effective and efficient method of recovering alumina. These acids are widely available and provide efficient alumina dissolving from coal waste products. Hydrochloric acid, in particular, is highly effective and selective for leaching alumina. However, during the leaching process, it might emit corrosive gases and fumes, prompting sufficient ventilation and safety precautions. Sulphuric acid is commonly utilised in alumina recovery due to its capability in dissolving aluminium from coal mine waste and CFA. However, because Sulphuric acid generates heat throughout the reaction, reaction conditions must be carefully monitored. For the best results when using Sulphuric acid, calcining coal mine waste and CFA, roasting, and a two-step acid-leaching method are required. The use of Sulphuric acid in leaching procedures not only reduces costs but also delivers reliable and efficient alumina extraction, making it a good choice in industrial settings. Nitric acid dissolves alumina and other components from coal waste materials with great selectivity and high recovery rates. However, because it is very corrosive and reactive, it poses difficulties in handling, storage, and safety. Furthermore, it is more expensive than other acid reagents. Because of their cost-effectiveness, availability, and high recovery rates, acid leaching using hydrochloric acid or Sulphuric acid is generally recommended for alumina recovery from coal mine waste and CFA. The use of microbial agents in bioleaching has the potential to increase alumina recovery from coal mine waste and CFA. Research in this field is somewhat restricted, primarily due to a scarcity of equipment and a limited number of investigations. Successful applications of Bacillus barbaricus and Acidithiobacillus ferrooxidans in bioleaching have proven effective for silica removal, alumina leaching, and extraction of other metals. Additionally, fungi, particularly Aspergillus niger, have shown promise in alumina recovery, although commercialisation is challenging given the specific conditions required for their effectiveness. Further research and development are essential in this domain to enhance bioleaching methods and evaluate their economic viability. The selection of a leaching reagent is influenced by factors such as the characteristics of the waste materials, the desired purity of the alumina product, and economic considerations. These innovative solutions not only have the potential to elevate alumina recovery but also contribute to fostering sustainable resource consumption and reducing waste output in the coal mining industry.

## 6 Technological advancements in mudstone and CFA for alumina recovery

Mudstone and CFA are potentially valuable materials for recycling in aluminium recovery, as extensive literature has conclusively demonstrated. Despite significant advancements in the utilisation of this coal-related waste, numerous barriers hinder its widespread application, including technological defects, economic weaknesses, policy and regulatory concerns, and public opinion barriers [121]. These products lack competitiveness and fail to attract sufficient investment to support continued development due to their low quality, high cost, and immature technology [91]. One viable strategy for waste management and sustainable resource utilisation is to recover alumina from CFA and mudstone. The following are some potential advancements that could drive futuristic technology in this field.

### 6.1 Advanced separation techniques

Efforts in research and development aimed at improving and refining separation methods present viable paths toward increasing alumina extraction from mudstone and CFA. The recovery process can be significantly enhanced by exploring more effective and discriminating separation techniques such as froth flotation, magnetic separation, and electrostatic separation. Prioritizing high recovery rates should be the focus of these approaches, while also aiming to simultaneously reduce energy consumption. Adopting cutting-edge separation science techniques and technologies offers the potential to extract higher recovery of alumina from these non-traditional sources, thereby improving resource efficiency and sustainability in the alumina industry.

### 6.2 Leaching and solvent extraction

The extraction efficiency of alumina from coal waste materials could be greatly enhanced through advancements in solvent extraction techniques and chemical leaching processes. This entails establishing benchmarks for the development of eco-friendly leaching agents and optimising extraction parameters to achieve high purity levels in the recovered alumina. By exploring innovative chemical compositions and refining operational parameters, scientists can strive to minimise environmental impact while optimising the production of premium alumina from coal waste streams. In addition to offering the potential for improved resource utilisation, this endeavour aligns with sustainability goals by reducing dependency on potentially hazardous conventional extraction techniques.

### 6.3 Nanotechnology applications

There is considerable potential for substantially improving the selectivity and adsorption capacity of materials employed in alumina recovery processes by employing nanomaterials and nanostructures. The application of nanotechnology in developing fresh catalysts and membranes designed for purification and separation tasks can enhance overall efficiency. Researchers can create adsorbents with a greater attraction to alumina molecules by exploiting the distinctive characteristics of nanoparticles, such as their increased surface area relative to volume and adaptable surface chemistry. This results in elevated extraction yields and enhanced purity.

### 6.4 Optimising alumina recovery: through integrated process design and automation

Optimising the entire process of alumina recovery requires the development of integrated process designs that incorporate several approaches for separation, extraction, and purification. Waste generation and energy consumption can be reduced by implementing process intensification strategies and integrating processes. Additionally, alumina recovery procedures are made more reliable, efficient, and safe with the use of modern automation. To efficiently respond to changing operational circumstances and optimise process parameters, real-time monitoring, predictive analytics, and adaptive control algorithms are integrated. When combined, these methods provide a thorough framework for optimising alumina recovery while maintaining operational efficacy and sustainability.

## 7 Conclusion

The preeminent and extensively employed approach for alumina recovery involves the process of heat treatment, specifically roasting, applied to alumina material before leaching. This method has been identified as the most practical and economically viable. The efficacy of this procedure relies upon meticulous optimisation of several parameters, including calcination temperature, leaching conditions, and roasting temperature, ensuring the maximization of alumina recovery. As ascertained by various scientists, optimal conditions encompass a calcination temperature within the range of 1000–1200 °C, a roasting temperature range of 750 °C–860 °C for 2 h, and leaching durations spanning 2–3 h. To achieve effective silica removal, the admixture of coal mine waste powder with sodium carbonate before roasting, in a ratio of 1:1 based on silica content in the sample, has manifested a realisation of alumina recovery exceeding 90 %. Another successfully identified method is biosulphate, which involves the utilisation of leaching reagents — sulphuric acid and ammonium sulphate or biosulphate, mixed in a ratio of 1/1. The experiment was conducted in an autoclave at a temperature of 220 °C, with a duration of 4 h and a solid-to-liquid ratio of 10:4. This resulted in a recovery efficiency exceeding 87.8 %. Upon consideration of factors such as affordability, accessibility, and efficiency, acid leaching using hydrochloric acid or sulphuric acid emerges as the optimal choice for alumina recovery. Conversely, the quality of aluminium sulphate generated during leaching with sulphuric acid remains unaffected by the presence of silica through this method. Consequently, prudent practice entails the integration of sodium carbonate with the alumina material before roasting, heat treatment, and eventual sulphuric acid leaching.

Microbial agents have proven highly effective in removing silica from alumina materials. While bioleaching methods yield an efficiency surpassing 80 %, they are characterised by a deliberate pace and inherent restrictions on commercialisation. Moreover, the enhancement of aluminium sulphate purity through purification techniques such as solvent extraction, precipitation and crystallisation become imperative. It is noteworthy that the specific techniques and parameters for separation and purification are contingent upon the metals involved, their concentrations, and the intended application of the metals post-purification. For efficient aluminium extraction and successful recovery of alumina, the pre-leaching heat treatment process is essential. Although this method consumes significant energy, it outperforms other techniques for recovering alumina from non-bauxite sources. A significant number of academic studies highlight the variation in alumina recovery from mudstone and CFA, depending on the composition and source of the material. As such, recovery circumstances vary according to sample origin, highlighting the critical requirement of thorough raw material characterisation before method development Although the majority of the material currently in publication focuses on using CFA for alumina recovery, it is important to understand that coal mine waste such as mudstone or coal gangue contains a sizable amount of alumina. To further research on alumina recovery from the above-indicated coal mine waste for commercialisation, significant investigation is required.

## Data availability statement

The data associated with my study have been deposited into a publicly available repository, and they are included in the article/supplementary material/referenced in the article.

## Ethics declaration

Review and approval by an ethics committee were not required for this study as there were no conflicts of interest.

## CRediT authorship contribution statement

**Amani Shilla:** Writing – review & editing, Writing – original draft. **Gershom Mwandila:** Supervision.

## Declaration of competing interest

The authors declare the following financial interests/personal relationships which may be considered as potential competing interests: AMANI MICCA SHILLA reports administrative support was provided by The 10.13039/501100007286Copperbelt University. If there are other authors, they declare that they have no known competing financial interests or personal relationships that could have appeared to influence the work reported in this paper.
